# Suicidal Thoughts and Behaviors and Their Associations With Transitional Life Events in Men and Women: Findings From an International Web-Based Sample

**DOI:** 10.2196/18383

**Published:** 2020-09-11

**Authors:** Alyssa Clare Milton, Tracey A Davenport, Frank Iorfino, Anna Flego, Jane M Burns, Ian B Hickie

**Affiliations:** 1 Brain and Mind Centre University of Sydney Camperdown Australia; 2 The Movember Foundation Melbourne Australia; 3 Faculty of Health Sciences The University of Sydney Sydney Australia

**Keywords:** suicidal ideation, suicide, suicide, attempted, men, women, sex differences, life change events, adaptation, psychological, health surveys

## Abstract

**Background:**

Although numerous studies have demonstrated sex differences in the prevalence of suicidal thoughts and behaviors (STB), there is a clear lack of research examining the similarities and differences between men and women in terms of the relationship between STB, transitional life events, and the coping strategies employed after experiencing such events when they are perceived as stressful.

**Objective:**

This study aims to examine the differences between men’s and women’s experiences of STB, sociodemographic predictors of STB, and how coping responses after experiencing a stressful transitional life event predict STB.

**Methods:**

A web-based self-report survey was used to assess the health and well-being of a voluntary community-based sample of men and women aged 16 years and older, living in Australia, Canada, New Zealand, the United Kingdom, and the United States, who were recruited using web-based social media promotion and snowballing.

**Results:**

In total, 10,765 eligible web-based respondents participated. Compared with men, a significantly greater proportion of women reported STB (*P*<.001) and endorsed experiencing a transitional life event as stressful (*P*<.001). However, there were no gender differences in reporting that the transitional life event or events was stressful for those who also reported STB. Significant sociodemographic adjusted risk factors of STB included younger age; identifying as a sexual minority; lower subjective social connectedness; lower subjective intimate bonds; experiencing a stressful transitional life event in the past 12 months; living alone (women only); not being in employment, education, or training (women only); suddenly or unexpectedly losing a job (men only); and experiencing a relationship breakdown (men only). Protective factors included starting a new job, retiring, having a language background other than English, and becoming a parent for the first time (men only). The results relating to coping after experiencing a self-reported stressful transitional life event in the past 12 months found that regardless of sex, respondents who reported STB compared with those who did not were less likely to engage in activities that promote social connections, such as talking about their feelings (*P<*.001). Coping strategies significantly explained 19.0% of the STB variance for men (*F*_16,1027_=14.64; *P*<.001) and 22.0% for women (*F*_16,1977_=36.45; *P*<.001).

**Conclusions:**

This research highlights multiple risk factors for STB, one of which includes experiencing at least one stressful transitional life event in the past 12 months. When individuals are experiencing such events, support from services and the community alike should consider using sex-specific or targeted strategies, as this research indicates that compared with women, more men *do nothing* when experiencing stress after a transitional life event and may be waiting until they experience STB to engage with their social networks for support.

## Introduction

### Background

Despite a considerable increase in global efforts to address suicidal thoughts and behaviors (STB), a corresponding decrease in the prevalence of death by suicide has not been observed [1]. STB are strong predictors of suicide deaths [2], contributing to substantial disability among individuals and having broader impacts on the individual’s social networks and the wider economy [3-5]. Suicide continues to be a significant public health concern [6], and improving our understanding of the factors associated with STB to mitigate risk, enhance health promoting behaviors, and evolve current support systems is clearly an ongoing need.

Men die by suicide significantly more frequently than women [7,8], although lifetime STB are higher among women than men [9]. This gender paradox is even more pronounced in high-income countries [8]. Furthermore, men in their middle years are at one of the highest risks for suicide in English-speaking, high-income countries such as Australia, Canada, the United Kingdom, and the United States [10-13].

Suicide and its relationship to transitional life events during adulthood is starting to receive increased attention in the literature [6,14]. A systematic review by Lui and Miller [6] found evidence of an association between negative life events, which they define as *life stressors*, and STB. Transitional life events can relate to one’s stage of development (such as retirement), historical events (such as war), and idiosyncratic factors (such as a change in an individual’s health status) [15]. To date, however, there has been an insufficient level of international research examining how transitional life events relating to different life stages predict STB in both men and women. This may be important, as such transitional life events can initiate a shift in the purpose and the direction of life [15], and unsuccessful transitions at certain stages may be associated with higher levels of psychological vulnerability. Indeed, transitional life events have the potential to impact individuals differently, with some individuals perceiving them as stressful (ie, a negative life event) and some not.

During transitional life events, an individual needs to use coping strategies, which can be adaptive or maladaptive, to adapt to the new stage in life [16,17]. In brief, maladaptive coping has been referred to as dysfunctional, regressive, or avoidant coping in the literature [18]. For example, Zuckerman and Gagne [19] highlight that maladaptive coping can involve disengagement, denial, blame, or self-punishment. It is hypothesized that these are key risk factors for STB. Adaptive coping, on the other hand, has often been defined as functional, transformative, or approach focused, where individuals use coping strategies such as problem solving, help seeking, and emotional expression in response to stressors [18]. Interestingly, a meta-analysis by Tamres et al [20] reported sex differences in coping, with women predominantly engaging in more adaptive or *expressive* types of coping strategies. How an individual copes (ie, in adaptive or maladaptive ways) if they perceive the life event as stressful may also be associated with STB. However, to our knowledge, comparing both adaptive and maladaptive coping strategies of men and women who have, or have not, experienced STB after experiencing a transitional life event has not been studied.

### Objectives

This substudy analyzes data collected in an international research project, the Global Health & Wellbeing Survey [21,22], and provides a unique opportunity to explore, on a larger scale, men’s and women’s experiences of STB and their associations—with a particular focus on transitional life events, how these events are perceived, and how an individual copes when experiencing these events.

The aims of this substudy are to assess (1) the differences between men and women in their experience of STB, transitional life events, and their perception of the event in the past 12 months; (2) the sociodemographic (including demographics, social connections, and transitional life events) predictors of STB in the past 12 months for men and women; and (3) the sex differences in the methods of coping with stressful transitional life events for those who have experienced STB compared with those who have not.

## Methods

### Design

A web-based survey methodology was used to assess the views of a voluntary community sample living in 5 target countries, including Australia, Canada, New Zealand, the United Kingdom, and the United States.

### Participants

The survey sample included men and women (aged 16 years and older) who reported that they had lived in 1 of the 5 target countries for the best part of the past 12 months.

### Procedure

The primary study received institutional ethics approval from the University of Sydney’s Human Research Ethics Committee (protocol number 2015/412). The open survey was tested before being hosted on the web from July 1, 2015, to December 11, 2015. A web-based advertising methodology was used to recruit respondents in the 5 target countries. Several strategies were used for survey dissemination and recruitment, including using multiple social media channels (eg, Facebook, Twitter, YouTube, and Instagram) [23]; layering of recruitment messages [24]; and passive snowballing via social media to spread study information through sharing, liking, and tweeting [23,25]. Both paid (via Facebook and YouTube, with budget set at Aus $10 [US $7.6] per participant, maximum spend of Aus $100,000 [US $76,400]) and free social media advertising (via a Facebook page, Twitter, and Instagram) were used. Recruitment targeting, based on age, sex, and region, was carried out through paid Facebook advertising channels to increase responses from harder-to-reach groups. This approach was supplemented by a traditional snowballing technique [26], in which respondents and wider networks forwarded the study to others. Respondents gave consent through the web and understood that they could cease participation at any time and that their responses were confidential and nonidentifiable. No incentives to participate were provided. Depending on participant answers, the survey took between 20 and 45 min to complete.

### Measures

Items in this substudy were extracted from the full Global Health & Wellbeing Survey*.* The number of questions completed by each participant varied depending on the skip pattern. The following are the areas of interest specific to this substudy: demographics, social connectedness, transitional life events, and STB.

#### Demographics

Demographic items included sex (response options: men vs women); age (responses collapsed into age groups 16-24 years, 25-44 years, 45-64 years, and ≥65 years); language background (language background other than English [LBOTE] vs English); living in a rural or remote location (responses collapsed into yes vs no); living arrangement (living alone vs not living alone); employment, education, and training status (responses collapsed into not in education, employment, or training [NEET] vs in education, employment, or training [EET]); and sexual orientation (responses collapsed into heterosexual vs lesbian, gay, bisexual, transsexual, queer, intersex, or asexual [LGBTQIA]).

#### Social Connectedness

Perceived social support and conflict in close relationships were measured by the 5-item Schuster’s Social Support and Conflict Scale [27] (Cronbach α=.71; n=8139). The 12 items on the care dimension of the Intimate Bond Measure [28] were used as an indicator of perceived care from one’s partner (Cronbach α=.96; n=8102).

#### Transitional Life Events

Respondents indicated whether or not they had experienced any of 7 specific transitional life events over the past 12 months, including finishing high school or secondary school, starting university or college, starting a new job, becoming a parent for the first time, suddenly or unexpectedly becoming unemployed, relationship breakdown, and retiring*.* Those who affirmed they had experienced one of these events were asked whether they found this experience stressful (*yes* or *no*). If *yes*, respondents were asked about their coping strategies. Items included a range of adaptive and maladaptive coping strategies (eg, *get professional help* or *become aggressive*).

#### STB

Respondents’ level of suicidality over the past 12 months was measured using the suicidal thoughts and acts subscale from the Psychiatric Symptom Frequency Scale (PSFS) [29], which was originally developed in the United Kingdom and has been used in both Australia and North America [30-33]. The subscale consists of 5 items concerning suicidal thoughts and acts that are each presented with dichotomous response options (*yes* and *no*). From the subscale, we can infer 4 different areas of suicidality: (1) suicidal thoughts, a desire to end one’s life without a specific plan; (2) suicide plans, formulating a strategy to end one’s life; (3) suicide attempts, nonfatal self-injurious behavior that includes some intent to end one’s life; and (4) STBs, the combination of an individual engaging in suicidal thoughts, plans, and/or attempts (Cronbach α=.82; n=8705).

### Analysis

Survey data were prepared and analyzed using IBM SPSS Statistics for Windows, version 22.0 (2013; IBM Corp). A 95% confidence level was used across all the analyses.

Chi-square tests were conducted to assess for significant differences between men and women in their experience of STB and transitional life events in the past 12 months (aim 1). Subsequently, Cramer V was calculated to determine the strength of the association. To adjust for type 1 error, a Holm-Bonferroni correction was applied.

Owing to the significant gender differences highlighted in aim 1, 2 logistic regression analyses (separately for men and women) were conducted to address aim 2. Following previously established procedures [34], 2 logistic regressions were run separately for men and women to identify sociodemographic (including demographics, social connectedness, specific transitional life events experienced in the past 12 months, and whether these life events were perceived as stressful) predictors of STB based on the PSFS.

To address aim 3, additional chi-square and Cramer V analyses were conducted to compare sex differences in coping after experiencing transitional life events for those who have experienced STB with those who have not (aim 3). For all analyses, no missing data were imputed, and 95% confidence levels were used. Again, a Holm-Bonferroni correction was applied to adjust for type 1 error. Owing to the noted sex differences, 2 additional linear regression analyses were used to examine the association between coping items and STB by sex. Missing data were excluded listwise. Regression model assumptions of linearity, homoscedasticity, independence, and normality (evaluated using standard residual-based diagnostic procedures) were met, with collinearity estimates among variables in both models being acceptable (tolerance >0.40; Durbin Watson >1.0).

### Ethical Standards

The authors assert that all procedures that contributed to this work complied with the ethical standards of the relevant national and institutional committees on human experimentation and with the Helsinki Declaration of 1975, as revised in 2008.

## Results

### Respondent Participation Rates and Characteristics

A total of 16,510 people were presented with the consent and eligibility screen. Of these, the total eligible sample was 10,765 respondents (10,765/16,510, 65.20%). Of those excluded, 75.30% (4326/5745) did not provide consent to participate and 12.36% (710/5745) were aged younger than 16 years.

In total, 31.11% (3349/10,765) of eligible participants were from Australia, followed by 18.00% (1938/10,765) from the United Kingdom, 17.54% (1888/10,765) from Canada, 17.07% (1838/10,765) from the United States, and 16.27% (1752/10,765) from New Zealand. The majority of the participants were women (6464/10,765, 60.05%), aged 45 to 64 years (3625/10,753, 33.71%), not living in rural or remote areas (6787/10,703, 63.41%), living with others (5781/7115, 81.25%), heterosexual (5923/7116, 83.23%), from an English-speaking background (5971/7484, 79.78%), and in paid EET (7220/9998, 72.21%). The details of participant characteristics are presented in Table 1, and a detailed breakdown of sociodemographic variables by sex and country are presented in Multimedia Appendices 1 and 2, respectively. Of the respondents who experienced at least one transitional life event in the past 12 months, 70.48% (3142/4458) reported that it was stressful.

### STB and Stressful Transitional Life Events

In the past 12 months, almost one-fourth of respondents in the full sample reported thinking about taking their own lives (2071/8708, 23.78%), 7.81% (680/8708) had made plans, and 3.03% (264/8708) had attempted to take their own life. Compared with men, a significantly greater proportion of women reported experiencing each of the PSFS items, including attempts (*P*≤.001), plans (*P*≤.001), and thoughts (*P=*.01). A large effect size (Cramer V ≥0.05) was seen for the item *attempted to take your own life.* All *P* values remained at the same level of significance after a Holm-Bonferroni correction was applied (see Multimedia Appendix 3 for a full breakdown of the PSFS results).

The frequency statistics of respondents’ experience of a major life event or events in the past 12 months and their responses are presented in Table 2 and address aim 1. About half (4450/9029, 49.29%) of all respondents experienced at least one of the 7 presented major life events. Of the respondents who experienced at least one major life event in the past 12 months, 70.45% (3135/4450) reported that it was stressful. Women were significantly more likely than men to endorse experiencing the major life event or events as stressful (*P*<.001). In total, 84.40% (1390/1647) of the respondents who had also experienced STB in the past 12 months reported the major life event or events as stressful. These results were not statistically different between genders. When considering people who had experienced at least one major life event in the past 12 months but reported no suicidal thoughts, significantly more women felt the major life event or events was stressful (women 65.58% [1069/1630] vs men 55.4% [546/986]; *P*<.001). All results remained the same after applying the Holm-Bonferroni correction.

**Table 1:** Participants’ sociodemographics by sex.

Variable		Full sample
**Sex (N=10,765), n (%)**		
	Male	4301 (39.95)
	Female	6464 (60.05)
**Age (years; n=10,753), n (%)**		
	16-24	2690 (24.99)
	25-44	2444 (22.71)
	45-64	3625 (33.68)
	≥65	1994 (18.53)
**Rural or remote (n=10,703), n (%)**		
	No	9990 (93.34)
	Yes	713 (6.66)
**EET^a^status (n=9998), n (%)**		
	No (not in education, employment, or training)	2778 (27.79)
	Yes (EET)	7220 (72.21)
**Living arrangements (n=7115), n (%)**		
	Live with others	5781 (81.25)
	Live alone	1334 (18.75)
**Language background (n=7484), n (%)**		
	English	5971 (79.78)
	Language background other than English	1513 (20.22)
**Sexual orientation (n=7116), n (%)**		
	Heterosexual	5923 (83.23)
	Lesbian, gay, bisexual, transsexual, queer, intersex, or asexual	1193 (16.77)
**Social connectedness, mean (SD)**		
	Intimate bonds (n=8102)	25.5 (9.5)
	Social support (n=8139)	9.3 (2.7)
**Transitional life event (yes), n (%)**		
	Became a parent for the first time (n=9035)	145 (1.60)
	Finished high school or secondary school (n=9034)	606 (6.71)
	Started university or college (n=9032)	799 (8.85)
	Started a new job (n=9037)	2153 (23.82)
	Suddenly or unexpectedly become unemployed (n=9033)	793 (8.77)
	Retired (n=9034)	598 (6.62)
	Relationship breakdown (n=9034)	2073 (22.95)
	Transitional life event perceived as stressful (n=4458)	3142 (70.48)

^a^EET: education, employment, or training.

**Table 2:** Frequency and experience of major life events, stress responses, and suicidal thoughts and behaviors.

Response			Total sample, n (%)		Men, n (%)		Women, n (%)		Men versus women						
									Chi-square (*df*)		*P* value		Cramer V		Holm-Bonferroni correction
**Experienced a major life event**															
	No	4579 (50.71)		1995 (43.57)		1633 (36.70)		44.3 (1)		<.001		0.07		<.001	
	Yes	4450 (49.29)		2584 (56.12)		2817 (63.30)		44.3 (1)		<.001		0.07		<.001	
**If yes, did you find this experiences stressful?**															
	No	1315 (29.55)		563 (34.48)		752 (26.70)		30.1 (1)		<.001		0.08		<.001	
	Yes	3135 (70.45)		1070 (65.52)		2065 (73.30)		30.1 (1)		<.001		0.08		<.001	
**Suicidal thoughts and behaviors (past 12 months)**															
	Stressful experience	1390 (84.40)		471 (84.11)		920 (84.56)		0.1 (1)		.80		0.01		.80	
	Experience not stressful	257 (15.60)		89 (15.89)		168 (15.44)		0.1 (1)		.80		0.01		.80	
**No suicidal thoughts and behaviors (past 12 months)**															
	Stressful experience	1630 (62.31)		561 (56.04)		1069 (66.19)		27.1 (1)		<.001		0.10		<.001	
	Experience not stressful	986 (37.69)		440 (43.96)		546 (33.81)		27.1 (1)		<.001		0.10		<.001	

### Predictors of STB

Adjusted odds ratios (AORs) and 95% CIs for the 2 subsamples (men and women) are presented in Table 3 (see Multimedia Appendix 4 results, including unadjusted risk ratios) and address aim 2. The models explained 24.0% and 31.0% of the variance for men and women, respectively (Nagelkerke *R*^2^ men=0.24; Nagelkerke *R*^2^ women=0.31).

The results presented in Table 3 show that men and women shared some similarities in relation to sociodemographic risks of STB. Specifically, those in younger age groups, particularly those aged 16 to 24 years compared with those aged 65 years or older (men: 16-24 years; AOR 2.89, 95% CI 1.96-4.26; *P<*.001 and women: 16-24 years; AOR 4.03, 95% CI 2.78-5.85; *P<*.001), those who identified as LGBTQIA (men: AOR 1.96, 95% CI 1.56-2.46; *P<*.001 and women: AOR 3.01, 95% CI 2.45-3.71; *P<*.001), and those who perceived a transitional life event they experienced in the past 12 months as stressful (men: AOR 1.38, 95% CI 1.15-1.67; *P=*.001 and women: AOR 1.55, 95% CI 1.34-1.79; *P<*.001), had significantly elevated AOR of STB. For men, significantly higher AOR of STB were observed for those who experienced a relationship breakdown (AOR 1.45, 95% CI 1.06-1.98; *P*=.02) and those who suddenly or unexpectedly became unemployed (AOR 1.63, 95% CI 1.16-2.29; *P=*.005). For women, identifying as NEET (AOR 1.67, 95% CI 1.31-2.15; *P<*.001) and living alone (AOR 1.38, 95% CI 1.11-1.71; *P=*.004) were associated with increased AOR of STB.

When considering protective factors, men and women had similar findings. Specifically both men and women had significantly lower AOR of STB if they identified as having a LBOTE (men: AOR 0.67, 95% CI 0.51-0.88; *P=*.004 and women: AOR 0.64, 95% CI 0.51-0.80; *P<*.001), reported higher levels of satisfaction with their social support (men: AOR 0.87, 95% CI 0.83-0.90; *P<*.001 and women: AOR 0.78, 95% CI 0.75-0.81; *P<*.001), higher intimate bonds ratings (men: AOR 0.96, 95% CI 0.95-0.98; *P<*.001 and women: AOR 0.98, 95% CI 0.97-0.99; *P<*.001), started a new job (men: AOR 0.73, 95% CI 0.55-0.97; *P*=.02 and women: AOR 0.80, 95% CI 0.65-1.00; *P=*.049), or retired (men: AOR 0.52, 95% CI 0.33-0.82; *P=*.004 and women: AOR 0.44, 95% CI 0.29-0.68; *P<*.001). Men who became a parent for the first time in the past 12 months had lower AOR of STB (AOR 0.30, 95% CI 0.10-0.86; *P=*.026).

**Table 3:** Adjusted odds ratios for men’s and women’s self-reported suicidal thoughts and behaviors by sociodemographic variables, including social connections and transitional life events (men, n=2667 and women, n=3826).

Participant characteristics			Suicidal thoughts and behaviors (Psychiatric Symptom Frequency Scale), adjusted odds ratio (95% CI)^a^	
			Men	Women
**Sociodemographics**				
	**Age bands (years) versus ≥65**			
		16-24	2.89 (1.96-4.26)^b^	4.03 (2.78-5.85)^b^
		25-44	1.97 (1.38-2.80)^b^	2.71 (1.89-3.89)^b^
		45-64	1.96 (1.46-2.64)^b^	1.81 (1.31-2.49)^b^
		≥65	1.00 (1.00-1.00)	1.00 (1.00-1.00)
	**Rural**			
		Yes	1.04 (0.72-1.50)	1.35 (0.98-1.85)
		No	1.00 (1.00-1.00)	1.00 (1.00-1.00)
	**Sexual orientation**			
		Lesbian, gay, bisexual, trans, queer, intersex, or asexual	1.96 (1.56-2.46)^b^	3.01 (2.45-3.71)^b^
		Heterosexual	1.00 (1.00-1.00)	1.00 (1.00-1.00)
	**Language background other than English**			
		Yes	0.67 (0.51-0.88)^b^	0.64 (0.51-0.80)^b^
		No	1.00 (1.00-1.00)	1.00 (1.00-1.00)
	**EET^c^**			
		No (not in education, employment, or training)	1.27 (0.98-1.63)	1.67 (1.31-2.15)^b^
		Yes (EET)	1.00 (1.00-1.00)	1.00 (1.00-1.00)
	**Living arrangements**			
		Live alone	1.27 (0.99-1.61)	1.38 (1.11-1.71)^b^
		Live with others	1.00 (1.00-1.00)	1.00 (1.00-1.00)
	**Social connectedness**			
		Intimate bonds	0.96 (0.95-0.98)^b^	0.98 (0.97-0.99)^b^
		Social support	0.87 (0.83-0.90)^b^	0.78 (0.75-0.81)^b^
**Transitional life events**				
		Became a parent for the first time	0.30 (0.10-0.86)^d^	0.66 (0.31-1.41)
		Finished high school or secondary school	1.07 (0.65-1.77)	0.96 (0.69-1.33)
		Started university or college	0.81 (0.54-1.21)	0.83 (0.62-1.10)
		Started a new job	0.73 (0.55-0.97)^d^	0.80 (0.65-1.00)^d^
		Suddenly or unexpectedly become unemployed	1.63 (1.16-2.29)^b^	1.24 (0.93-1.66)
		Retired	0.52 (0.33-0.82)^b^	0.44 (0.29-0.68)^b^
		Relationship breakdown	1.45 (1.06-1.98)^d^	0.93 (0.73-1.18)
Transitional life event perceived as stressful			1.38 (1.15-1.67)^b^	1.55 (1.34-1.79)^b^

^a^Adjusted for all variables.

^b^Significant at 99% confidence level.

^c^EET: education, employment, or training.

^d^Significant at 95% confidence level.

### Coping After Experiencing a Stressful Life Event

There were 5 areas consistently identified by respondents as top coping strategies, irrespective of gender. These included sleeping too much or too little (2279/3130, 72.81% of all respondents), talking to someone about their feelings (2177/3132, 69.51% of all respondents), eating more or eating less (2069/3130, 66.10% of all respondents), isolating themselves (1795/3138, 57.20% of all respondents), and talking to someone for advice (1790/3129, 57.21% of all respondents). Table 4 presents further frequency data relating to men’s and women’s coping responses after experiencing a transitional life event perceived as stressful by self-reported STB in the past 12 months.

**Table 4:** Frequency of men’s and women’s coping responses after experiencing a stressful transitional life event for participants who did and did not report suicidal thoughts and behaviors.

Response based on stressful life experience	Suicidal thoughts and behaviors (Psychiatric Symptom Frequency Scale)				
	Men		Women		
	No	Yes		No	Yes
Sample size, range	561-563	467-471		1063-1071	916-919
Became aggressive (% yes)	14.59	27.66		8.71	19.28
Bossy or inflexible or angry (% yes)	28.29	37.58		29.78	43.96
Eat more or less (% yes)	47.51	66.81		62.90	80.07
Engage in spiritual activity (% yes)	31.49	27.66		32.02	30.50
Got professional help (% yes)	25.09	47.66		23.20	46.90
Increased tobacco or alcohol or drugs (% yes)	26.82	43.31		20.02	37.11
Isolated self (% yes)	43.59	75.16		40.24	76.17
Overdo activities (% yes)	20.28	25.32		26.29	32.86
Sleep too much or too little (% yes)	60.85	80.64		65.29	84.31
Spend time with friends or loved ones (% yes)	32.74	22.51		39.70	25.16
Work less or more (% yes)	31.32	42.34		27.81	46.51
Take more risks (% yes)	20.82­­	35.46		14.41	29.27
Talk to someone about feelings (% yes)	64.48	66.38		75.51	67.14
Talk to someone for advice (% yes)	50.36	56.60		59.31	59.37
Do nothing (% yes)	23.67	33.05		17.57	33.33
Other (% yes)	13.01	16.70		12.32	18.45

An in-depth chi-square and Cramer V analyses using a Bonferroni correction, addressing aim 3, showed that there were multiple sex differences in coping after experiencing a transitional life event that was perceived by the individual as stressful (see Multimedia Appendix 5 for a full breakdown of results). Regardless of whether a respondent experienced STB in the past 12 months or not, women reported significantly higher rates of eating more or eating less (no STB: 62.90% [women] vs 47.51% [men]; *P<*.001 and STB: 80.07% [women] vs 66.81% [men]; *P<*.001) and lower rates of overdoing activities (no STB: 26.29% [women] vs 20.28% [men]; *P<*.01 and STB: 32.86% [women] vs 25.32% [men]; *P<*.01). Conversely, compared with women, men reported significantly higher rates of becoming aggressive (no STB: 8.71% [women] vs 14.59% [men]; *P<*.001 and STB: 19.28% [women] vs 27.66% [men]; *P<*.001); taking more risks (no STB: 14.41% [women] vs 20.82% [men]; *P<*.01 and STB: 29.27% [women] vs 35.46% [men]; *P=*.02); and increasing their use of tobacco, alcohol, or other drugs (no STB: 20.02% [women] vs 26.82% [men]; *P<*.01 and STB: 37.11% [women] vs 43.31% [men]; *P=*.03). Of these, eating more or eating less and becoming aggressive remained significant after adjusting for multiple comparisons using a Bonferroni correction.

For those who did not experience STB, women reported significantly higher rates of talking to someone about their feelings (75.51% [women] vs 64.48% [men]; *P<*.001), spending more time with friends and loved ones (39.70% [women] vs 32.74% [men]; *P<*.01), and talking to someone for advice (59.31% [women] vs 50.36% [men]; *P<*.01); however, there were no differences between men and women for these variables if the respondent had also experienced STB in the past 12 months. Finally, men reported higher rates of doing nothing (17.57% [women] vs 23.67% [men]; *P<*.01) when they had not experienced STB in the past 12 months; however, there were no differences between men and women for this variable if respondents did experience STB.

When comparing all participant’s coping responses after experiencing a transitional life event that was perceived as stressful, the respondents who did report STB were significantly more likely to engage in detrimental activities such as becoming aggressive (22.10% [STB] vs 10.74% [no STB]; *P<*.001); becoming bossy or inflexible or angry with others (41.80% [STB] vs 29.26% [no STB]; *P<*.001); eating more or eating less (75.58% [STB] vs 57.60% [no STB]; *P<*.001); increasing their use of tobacco, alcohol, or other drugs (39.21% [STB] vs 22.37% [no STB]; *P<*.001); isolating themselves (75.83% [STB] vs 41.40% [no STB]; *P<*.001); overdoing activities (30.31% [STB] vs 24.22% [no STB]; *P<*.001); sleeping too much or too little (83.07% [STB] vs 63.76% [no STB]; *P<*.001); working more or working less (45.10% [STB] vs 29.02% [no STB]; *P<*.001); taking more risks (31.37% [STB] vs 16.62% [no STB]; *P<*.001); and doing nothing (33.24% [STB] vs 19.67% [no STB]; *P<*.001). Furthermore, this group was significantly less likely to engage in the potentially helpful activities of spending more time with friends and loved ones (24.26% [STB] vs 37.30% [no STB]; *P<*.001) and talking to someone about their feelings (66.88% [STB] vs 71.71% [no STB]; *P<*.01). However, they were also significantly more likely to get professional help (47.16% [STB] vs 23.85% [no STB]; *P<*.001).

Table 5 shows 2 linear regression models, which present data on how the different types of coping strategies (after experiencing a transitional life event perceived as stressful) explained the variance in STB for men and women (aim 3). For men, the regression model significantly explained 19.0% of the variance in STB (*F*_16,1027_=14.64; *P*<.001; *R*^2^ adjusted=0.19), with 7 variables accounting for this variance at a 95% confidence level. In order of effect size, this included isolating self (ß=.21; *P*<.001), getting professional help (ß=.20; *P*<.001), doing nothing (ß=.09; *P=*.004), becoming aggressive (ß=.09; *P=*.004), sleeping too much or too little (β=.09; *P*=.005), increasing alcohol and other drug use (β=.06; *P=*.036), and other (β=.06; *P=*.048). For women, the regression model significantly explained 22.0% of the variance in STB (*F*_16,1977_=36.45; *P*<.001; *R*^2^ adjusted=0.22), with 8 significant variables. In order of effect size, this included isolating self (β=.22; *P*<.001), getting professional help (β=.20; *P*<.001), doing nothing (β=.12; *P*<.001), increasing alcohol and other drug use (β=.08; *P*<.001), sleeping too much or too little (β=.07; *P=*.002), becoming aggressive (β=.05; *P=*.015), taking more risks (β=.05; *P=*.027), and working less or working more (β=.04; *P=*.036).

**Table 5:** Linear regression of coping responses after experiencing a stressful transitional life event as predictors of suicidal thoughts and behaviors by sex.

Variable		Linear regression (*t*)		*P* value		β		95% CI	
**STB^a^ in men**									
	Became aggressive		2.87		.004		.09		0.04 to 0.19
	Bossy or inflexible or angry		−0.72		.47		−.02		−0.09 to 0.04
	Eat more or less		1.61		.11		.05		−0.01 to 0.11
	Spiritual activity		−1.78		.08		−.05		−0.12 to 0.01
	Got professional help		6.10		<.001		.20		0.14 to 0.27
	Increased tobacco or alcohol or drugs		2.10		.036		.06		0.00 to 0.13
	Isolated self		6.38		<.001		.21		0.15 to 0.28
	Overdo activities		−0.56		.55		−.02		−0.09 to 0.05
	Sleep too much or too little		2.84		.005		.09		0.03 to 0.16
	Spend time with friends or loved ones		−1.43		.15		−.04		−0.12 to 0.02
	Work less or more		−1.16		.25		−.04		−0.10 to 0.03
	Take more risks		1.60		.11		.05		−0.01 to 0.12
	Talk to someone about feelings		0.81		.42		.03		−0.04 to 0.11
	Talk to someone for advice		−0.51		.61		−.02		−0.09 to 0.05
	Do nothing		2.90		.004		.09		0.03 to 0.17
	Other		1.98		.048		.06		0.00 to 0.16
**STB in women**									
	Became aggressive		2.44		.015		.05		0.01 to 0.14
	Bossy or inflexible or angry		0.87		.38		.02		−0.02 to 0.06
	Eat more or less		1.92		.055		.04		0.00 to 0.09
	Spiritual activity		−1.48		.14		−.03		−0.08 to 0.01
	Got professional help		8.91		<.001		.20		0.17 to 0.26
	Increased tobacco or alcohol or drugs		3.98		<.001		.08		0.05 to 0.14
	Isolated self		9.47		<.001		.22		0.17 to 0.27
	Overdo activities		−1.38		.17		−.03		−0.08 to 0.01
	Sleep too much or too little		3.06		.002		.07		0.03 to 0.13
	Spend time with friends or loved ones		−1.55		.12		−.03		−0.08 to 0.01
	Work less or more		2.10		.036		.04		0.00 to 0.09
	Take more risks		2.22		.027		.05		0.01 to 0.11
	Talk to someone about feelings		−1.62		.11		−.04		−0.10 to 0.01
	Talk to someone for advice		−0.41		.68		−.01		−0.06 to 0.04
	Do nothing		5.27		<.001		.12		0.09 to 0.19
	Other		1.82		.06		.04		0.00 to 0.11

^a^STB: suicidal thoughts and behaviors.

## Discussion

### Principal Findings

This study describes an analysis of men’s and women’s experience of transitional life events, STB, and coping strategies, with data being gathered in the largest known international web-based survey in this area. In keeping with previous literature, the proportion of women reporting STB was significantly larger than the number of men reporting STB [35,36]. Following this pattern, women also reported at a significantly higher rate that the transitional life event (or events) they experienced was stressful, compared with men. These findings may suggest that the experience or the perception of stress is different between men and women, as highlighted in previous research [37]. Moreover, the results may indicate that women have more proactive coping strategies when faced with stressful transitional life events. This is considering the finding that women not experiencing STB were more likely to engage with their social networks when coping with life events they perceived as stressful. To some extent, this rationale supports meta-analytic research that women predominantly engage in more adaptive or *expressive* types of coping strategies [20]. However, in this study, there were no sex differences in reports of feeling stressed once experiencing both a transitional life event and STB. Furthermore, the sex differences in *expressive* style coping after a stressful transitional life event (specifically, talking to someone about feelings, spending time with friends or loved ones, and talking to someone for advice) were present when individuals were not experiencing STB but dissipated once individuals reported STB. Therefore, this study may extend previous meta-analytic findings [20]. Namely, it is possible that women articulate their perceived feelings of stress to others at an earlier stage (ie, when they are not experiencing STB), whereas men might be less likely to seek support from others until they move toward a crisis point.

The results from the logistic regression indicated that both men and women who reported experiencing a stressful transitional life event in the past 12 months had higher AOR of STB. The subsequent linear regression highlighted some potential warning signs for STB, most of which have been identified as predictors of future STB and are among the warning signs for many international guidelines [38]. This included becoming aggressive; self-isolating behavior; changes in sleep; and increased use of tobacco, alcohol, or other drugs for all participants, irrespective of their sex. For women specifically, warning signs also included increased risk taking and changes in work behaviors. Previous research has demonstrated a relationship between suicide and substance abuse [35,39], sleep disturbances [40], and social isolation [41]. This study adds to the literature by highlighting some identifiable signs that individuals experiencing STB engage in after going through a transitional life event (or events) they perceive as stressful.

These warning signs of STB, after experiencing a transitional life event, may be compounded when considering the chi-square finding that both men and women who reported STB, compared with those who did not, were significantly less likely to engage in potentially helpful coping strategies (eg, spending more time with friends and loved ones and talking to someone about one’s feelings). These strategies may help buffer a person against distress. Furthermore, it was found that respondents experiencing STB reported higher rates of accessing professional help compared with those who did not report STB, and seeking professional help significantly explained the variance in STB for men and women. Despite this, more than half of all the participants with STB did not seek help. Indeed, one-third of the participants who reported STB (33.2%) said they *did nothing* to cope after a stressful transitional life event. This explained the variance in STB for both sexes in the linear regression. Although research has consistently reported that men are less likely to seek help than women [42], this research found no differences in seeking professional help between men and women when experiencing STB. Notably, seeking professional help significantly explained the variance in STB in both men and women. Women, however, were more likely to seek professional help when experiencing stress but were not reporting STB. Again, this may indicate that men are waiting until they head toward a crisis point to seek help. This delay seen in men toward help seeking may be because of self-stigma, a wish to handle the problem on one’s own, and a low perceived need for care [42].

Other significant findings include that a majority of sociodemographic variables that resulted in higher or lower AOR of STB—such as identifying as LGBTQIA, NEET (women only), suddenly or unexpectedly becoming unemployed (men only), living alone (women only), satisfaction with intimate bonds, satisfaction with social support, starting a new job, becoming a parent for the first time (men only), and relationship breakdown (men only)—arguably relate to an individual’s level of social connectedness, or lack thereof. In the literature, social isolation and the absence of social support are established correlates of suicide risk, with O’Conner and Nock [43] suggesting that it is a vital component of contemporary models of suicidal behavior. Furthermore, the higher rates of STB among these various subgroups [44-48] could be attributed to disruptions or distress related to not only their social connectedness but also connectedness more broadly to include their job or perceived sense of purpose, which would be consistent with existing models that characterize the development of strong STB [49].

### Implications for Policy and Practice

When taking all these factors together, these findings emphasize the importance of promoting more helpful coping strategies after experiencing a stressful transitional life event. For example, men were more likely to engage in maladaptive coping strategies associated with completed suicides, such as substance abuse [35,39]. Health services and campaigns should focus on helping men recognize and respond to life stressors earlier. This includes equipping them with the skills to identify triggers and early warning signs of stress so that they can take appropriate action, seek help, and engage in more adaptive coping strategies earlier. This is also important for women, particularly given the finding that increased use of tobacco, alcohol, or other drugs was associated with STB for women only. Overall, a greater understanding in the community of these potential warning signs of STB in people who have experienced a stressful transitional life event might also improve the identification, response, and provision of support.

In addition, in accordance with other Australian research recommendations [50], enhancing social connectedness in the general community is crucial, particularly given the findings that isolating oneself as a coping strategy after experiencing a stressful transitional life event explained variance in STB for both men and women. Additional focus should also be placed on supporting and tailoring services to meet the needs of more vulnerable groups highlighted in this research, such as young people, people who identify as LGBTQIA, people experiencing employment-related concerns, people with lower satisfaction toward their social support and intimate bonds, men who experience relationship breakdown, and women who live alone. Overall, interventions that target improving community support and social connectedness should be considered in health services, educational institutions, and workplace settings, particularly after someone experiences a stressful transitional life event. Research suggests that augmentation of social connectedness through not only traditional face-to-face methods but also digital mental health supports should be considered [50,51], particularly given that these can improve access for more socially isolated individuals [52].

### Strengths and Limitations

A key strength of this study is that it is one of the largest samples to date, providing data on transitional life events, coping strategies, and multiple factors related to suicide risk. It is, however, not without limitations—many of which are outlined in the main reports’ executive summary, including the nonepidemiological nature of web-based research that holds the potential for avidity and internet bias [21]. Indeed, in this study, suicidal thoughts were common, with 23.8% of the total sample having thought about taking their own life in the past 12 months. Furthermore, suicide plans and attempts over the past 12 months were reported by 7.8% and 3.0% of the respondents, respectively. These rates are far higher than those reported in the literature, with the World Health Organization’s 12-month prevalence estimates being at 2.0% for suicidal thoughts, 0.6% for plans, and 0.3% for attempts within developed countries [53]. It is also possible that those with a greater interest in the subject matter were more likely to participate in this research. This research still provides valuable insights as the interactions between variables, not merely the statistical frequencies, provide meaningful information. However, a selection bias could mean that the findings relating to associations between life events, coping mechanisms, and STB may be more specific to higher risk groups. For this reason, we also did not compare differences across countries, although countries presented similar sociodemographics (Multimedia Appendix 2).

Additional limitations of this substudy are as follows. First, the brief screener for STB via the PSFS relies on binary responses and, thus, may be less sensitive to subtle response variances. Second, known predictors of suicide, such as mental ill health, alcohol, and/or other substance misuse [38], were also not considered in the multinomial regression, as we focused on sociodemographic factors. It is acknowledged that their inclusion would influence the results. Furthermore, only 7 transitional life events were considered in the Global Health & Wellbeing Survey. Other transitional life events may warrant further research (such as commencing a long-term relationship, getting married, and death of a loved one); however, because of the sheer scale of the survey, not all transitional life events could be included. In addition, some transitional life events, specifically experiencing a relationship breakdown and suddenly or unexpectedly becoming unemployed, are acknowledged to be far more of a negative experience than others included in this survey, which may have influenced the results. These were included, however, as they are becoming far more common in the included target countries, nearly one-fourth (22.9%) of the entire sample had experienced a relationship breakdown in the past 12 months. The research also relied on self-report, which asked participants to recall the transitional life events experienced and STB over the past 12 months. This lengthy recall period is a common issue in research [6]. Interview-based approaches instead of self-report checklists may allow for greater resolution in the dating of an event and provide further depth of assessment in the relationship between stressors and STB [6]. However, interview-based research has also been shown to limit the disclosure of sensitive items compared with web-based approaches, particularly for men and people not in education, training, or employment [54]. Thus, the web-based survey approach used in this study may allow for a greater level of disclosure of sensitive information, such as STB.

### Conclusions

Although nonepidemiological in nature, the considerable size of the Global Health & Wellbeing Survey provides some key insights into the international landscape for men and women, with good representation from minority subpopulations. Multifaceted approaches toward providing support to individuals experiencing STB after experiencing a stressful transitional life event is crucial. Essential to this includes risk mitigation through systematic identification and assessment [55] and a greater emphasis on health promoting behaviors and coping mechanisms within communities to strengthen resilience. When designing interventions, services should consider using sex-specific or targeted strategies to inform both the early identification of warning signs relating to STB and the provision of effective early intervention to mitigate the immediate risk and long-term impact of STB [56]. Beyond traditional interventions, these results show that enhancing social connectedness and reducing marginalization may be vital. This is especially relevant for groups at risk of marginalization, including those who had lower satisfaction with their social support and intimate bonds and specific populations, such as young people, people who identify as LGBTQIA, and have employment-related issues.

## Appendix

Multimedia Appendix 1 Participant socio-demographics for the full sample and by sex.

Multimedia Appendix 2 Participant socio-demographics by country.

Multimedia Appendix 3 Frequency and experience of suicidal thoughts and behaviours.

Multimedia Appendix 4 Odds and adjusted odds ratios for men and women’s self-reported suicidal thoughts and behaviors by sociodemographic variables including social connection and transitional life events (Men n=2667; Women n=3826).

Multimedia Appendix 5 Frequency of men and women’s responses based on stressful transitional life event experience for participants who did and did not report suicidal thoughts and behaviours (PSFS).

## Abbreviations

AOR: adjusted odds ratio
BMC: Brain and Mind Centre
EET: in education, employment, or training
LBOTE: language background other than English
LGBTQIA: lesbian, gay, bisexual, transsexual, queer, intersex, or asexual
NEET: not in education, employment, or training
PSFS: Psychiatric Symptom Frequency Scale
STB: suicidal thoughts and behaviors
