# Supplementary material for: Suicidal Thoughts and Behaviors and Their Associations With Transitional Life Events in Men and Women: Findings From an International Web-Based Sample
Source: JMIR Ment Health. 2020 Sep 11;7(9):e18383. doi: 10.2196/18383 (PMC7519425; doi:10.2196/18383)
Supplement: Multimedia Appendix 1 [file mental_v7i9e18383_app1.docx]

## Multimedia Appendices

Multimedia Appendix 1. *Participant socio-demographics for the full sample and by sex*

|  |  | **Full sample** | | **Females** | | **Males** | |
| --- | --- | --- | --- | --- | --- | --- | --- |
| **Variable** |  | **n** | **%** | **n** | **%** | **n** | **%** |
| **Gender** | N | 10,765 |  | 6,464 |  | 4,301 |  |
|  | *Male* | 4,301 | 40.0 | - | - | 4,301 | 100.0 |
|  | *Female* | 6,464 | 60.0 | 6,464 | 100.0 | - | - |
| **Age (years)** | N | 10,753 |  | 6,457 |  | 4,296 |  |
|  | *16 to 24* | 2,690 | 25.0 | 1,909 | 29.6 | 781 | 18.2 |
|  | *25 to 44* | 2,444 | 22.7 | 1,513 | 23.4 | 931 | 21.7 |
|  | *45 to 64* | 3,625 | 33.7 | 2,070 | 32.1 | 1,555 | 36.2 |
|  | *65+* | 1,994 | 18.5 | 965 | 14.9 | 1,029 | 24.0 |
| **Rural/ remote** | N | 10,703 |  | 6,430 |  | 4,273 |  |
|  | *No* | 9,990 | 93.3 | 6022 | 93.7 | 3,968 | 92.9 |
|  | *Yes* | 713 | 6.7 | 408 | 6.3 | 305 | 7.1 |
| **Education, employment and training (EET) status** | N | 9,998 |  | 5,927 |  | 4,071 |  |
|  | *No (NEET)* | 2,778 | 27.8 | 1,444 | 24.4 | 1,334 | 32.8 |
|  | *Yes (EET)* | 7,220 | 72.2 | 4,483 | 75.6 | 2,737 | 67.2 |
| **Living arrangements** | N | 7,115 |  | 4,232 |  | 2,883 |  |
|  | *Live alone* | 5,781 | 18.7 | 759 | 17.9 | 575 | 19.9 |
|  | *Do not live alone* | 1,334 | 81.3 | 3,473 | 82.1 | 2,308 | 80.1 |
| **Language Background** | N | 7,484 |  | 4,454 |  | 3,030 |  |
|  | *English* | 5,971 | 79.8 | 3,540 | 79.5 | 2,431 | 80.2 |
|  | *Language Background Other Than English (LBOTE)* | 1,513 | 20.2 | 914 | 20.5 | 599 | 19.8 |
| **Sexual orientation** | N | 7,116 |  | 4,233 |  | 2,883 |  |
|  | *Heterosexual* | 5,923 | 83.2 | 3,593 | 84.9 | 2,330 | 80.8 |
|  | *LGBTQIA* | 1,193 | 16.8 | 640 | 15.1 | 553 | 19.2 |
| **Social connectedness** |  | **N** | **Mean (SD)** | **N** | **Mean (SD)** | **N** | **Mean (SD)** |
|  | *Intimate Bonds (IBM)* | 8,102 | 25.5 (9.5) | 4,845 | 25.9 (9.4) | 3,257 | 25.0 (9.7) |
|  | *Social Support (SSSC)* | 8,139 | 9.3 (2.7) | 4,870 | 9.2 (2.8) | 3,269 | 9.3 (2.6) |
| **Transitional Life Event** |  | n | % | n | % | n | % |
|  | *Became a parent for the first time (Yes)* | 145 | 1.6 | 84 | 1.6 | 61 | 1.7 |
|  | *Finished high school/secondary school (Yes)* | 606 | 6.7 | 419 | 7.8 | 187 | 5.1 |
|  | *Started university/college (Yes)* | 799 | 8.8 | 529 | 9.8 | 270 | 7.4 |
|  | *Started a new job (Yes)* | 2,153 | 23.8 | 1,414 | 26.2 | 739 | 20.3 |
|  | *Suddenly or unexpectedly become unemployed (Yes)* | 793 | 8.8 | 462 | 8.6 | 331 | 9.1 |
|  | *Retired (Yes)* | 598 | 6.6 | 318 | 5.9 | 280 | 7.7 |
|  | *Relationship breakdown (Yes)* | 2,073 | 22.9 | 1,352 | 25.0 | 721 | 19.9 |
|  | *Transitional life event perceived as stressful* | 3,142 | 70.5 | 2,068 | 73.3 | 1,074 | 65.6 |
|  | *Transitional life event not perceived as stressful* | 1,316 | 29.5 | 753 | 26.7 | 563 | 34.4 |

Multimedia Appendix 2. *Frequency and experience of suicidal thoughts and behaviours*

| **PSFS items** |  | **Total Sample** | **Men** | **Women** | **χ2 (Men vs Women)** | ***P*** | **CV^a^** | **HB^b^** |
| --- | --- | --- | --- | --- | --- | --- | --- | --- |
| **In the past 12 months have you…** | N | 8,708 | 3,491 | 5,217 |  |  |  |  |
| *Felt that life is hardly worth living?* | (% Yes) | 30.8 | 27.2 | 33.3 | 37.09 | <.001 | .07 | <.001 |
| *Thought that you really would be better off dead?* | (% Yes) | 25.1 | 21.7 | 27.4 | 36.14 | <.001 | .06 | <.001 |
| *Thought about taking your own life?* | (% Yes) | 23.8 | 22.4 | 24.7 | 6.13 | .01 | .01 | .01 |
| *Made plans to take your own life?* | (% Yes) | 7.8 | 6.5 | 8.7 | 13.79 | <.001 | .04 | <.001 |
| *Attempted to take your own life?* | (% Yes) | 3.0 | 1.9 | 3.8 | 24.52 | <.001 | .05 | <.001 |
| *STB (Total PSFS)* | (% reporting any STB) | 30.7 | 28.3 | 32.3 | 15.87 | <.001 | .04 | <.001 |

1. Cramer’s V
2. Holm-Bonferroni correction

Multimedia Appendix 3. *Odds and adjusted odds ratios for men and women’s self-reported suicidal thoughts and behaviours by socio-demographic variables including social connection and transitional life events (Men n= 2,667; Women n= 3,826)*

|  | **PSFS** | | | |
| --- | --- | --- | --- | --- |
|  | **Women** | | **Men** | |
|  | **OR [95% CI]** | **Adjusted OR [95% CI]**^a^ | **OR [95% CI]** | **Adjusted OR [95% CI]**^a^ |
| **Socio-demographic characteristics** |  |  |  |  |
| Age-bands (years) *vs 65+* |  |  |  |  |
| *16 - 24* | 2.92 | 4.03 | 2.56 | 2.89 |
|  | [2.46-3.47]^b^ | [2.78-5.85]^b^ | [2.14-3.05]^b^ | [1.96-4.26]^b^ |
| *25 - 44* | 2.05 | 2.71 | 1.83 | 1.97 |
|  | [1.71-2.45]^b^ | [1.89-3.89]^b^ | [1.52-2.20]^b^ | [1.38-2.80]^b^ |
| *45 - 64* | 1.69 | 1.81 | 1.68 | 1.96 |
|  | [1.41-2.03]^b^ | [1.31-2.49]^b^ | [1.41-2.00]^b^ | [1.46-2.64]^b^ |
| *65+* | 1.00 | 1.00 | 1.00 | 1.00 |
| Rural |  |  |  |  |
| *Yes* | 1.08 | 1.35 | 1.03 | 1.04 |
|  | [0.93-1.26] | [0.98-1.85] | [0.84-1.26] | [0.72-1.50] |
| *No* | 1.00 | 1.00 | 1.00 | 1.00 |
| Sexual orientation |  |  |  |  |
| *Lesbian, Gay, Bisexual, Trans, Queer, Intersex, Asexual*  *(LGBTQIA)* | 2.16 | 3.01 | 1.78 | 1.96 |
|  | [1.98-2.35]^b^ | [2.45-3.71]^b^ | [1.59-2.01]^b^ | [1.56-2.46]^b^ |
| *Heterosexual* | 1.00 | 1.00 | 1.00 | 1.00 |
| Language background other than English (LBOTE) |  |  |  |  |
| *Yes* | 0.93 | 0.64 | 0.85 | 0.67 |
|  | [0.83-1.04] | [0.51-0.80]^b^ | [0.72-0.995]^c^ | [0.51-0.88]^b^ |
| *No* | 1.00 | 1.00 | 1.00 | 1.00 |
| Employment, education or training |  |  |  |  |
| *No (NEET)* | 1.09 | 1.67 | 1.04 | 1.27 |
|  | [0.99-1.21] | [1.31-2.15]^b^ | [0.92-1.16] | [0.98-1.63] |
| *Yes (EET)* | 1.00 | 1.00 | 1.00 | 1.00 |
| Living arrangements |  |  |  |  |
| *Live alone* | 1.01  [0.90-1.13] | 1.38  [1.11-1.71]^b^ | 1.28  [1.12-1.46]^b^ | 1.27  [0.99-1.61] |
|  |  |  |  |  |
| *Live with others* |  | 1.00 |  | 1.00 |
| Social connectedness |  |  |  |  |
| *Intimate Bonds (IBM)* | - | 0.98 | - | 0.96 |
|  |  | [0.97-0.99]^b^ |  | [0.95-0.98]^b^ |
| *Social Support (SSSC)* | - | 0.78 | - | 0.87 |
|  |  | [0.75-0.81]^b^ |  | [0.83-0.90]^b^ |
| Transitional life events |  |  |  |  |
| *Became a parent for the first time* | 0.92 | 0.66 | 0.75 | 0.30 |
|  | [0.65-1.30] | [0.31-1.41] | [0.46-1.25] | [0.10-0.86]^c^ |
| *Finished high school/secondary school* | 1.59 | 0.96 | 1.45 | 1.07 |
|  | [1.43-1.78]^b^ | [0.69-1.33] | [1.20-1.75]^b^ | [0.65-1.77] |
| *Started university/college* | 1.38 | 0.83 | 1.36 | 0.81 |
|  | [1.24-1.54]^b^ | [0.62-1.10] | [1.15-1.61]^b^ | [0.54-1.21] |
| *Started a new job* | 1.21 | 0.80 | 1.22 | 0.73 |
|  | [1.11-1.31]^b^ | [0.65-1.00]^c^ | [1.08-1.37]^b^ | [0.55-0.97]^c^ |
| *Suddenly or unexpectedly become unemployed* | 1.51 | 1.24 | 1.80 | 1.63 |
|  | [1.35-1.68]^b^ | [0.93-1.66] | [1.58-2.05]^b^ | [1.16-2.29]^b^ |
| *Retired* | 0.64 | 0.44 | 0.72 | 0.52 |
|  | [0.51-0.80]^b^ | [0.29-0.68]^b^ | [0.57-0.93]^b^ | [0.33-0.82]^b^ |
| *Relationship breakdown* | 1.88 | 0.93 | 2.11 | 1.45 |
|  | [1.74-2.03]^b^ | [0.73-1.18] | [1.91-2.34]^b^ | [1.06-1.98]^c^ |
| Transitional life event perceived as stressful | 1.96 | 1.55 | 2.71 | 1.38 |
|  | [1.71-2.26]^b^ | [1.34-1.79]^b^ | [2.22-3.31]^b^ | [1.15-1.67]^b^ |
| **Nagelkerke R^2^** |  | 0.31 |  | 0.24 |

^a^ adjusted for all variables.

^b^ significant at a 99% confidence level.

^c^ significant at a 95% confidence level.

Multimedia Appendix 4. *Frequency of men and women’s responses based on stressful transitional life event experience for participants who did and did not report suicidal thoughts and behaviours (PSFS)*

|  | | | **Suicidal thoughts and behaviours PSFS** | | | | | | | | | **Suicidal thoughts and behaviours for total sample** | | | | **Did not experience suicidal thoughts and behaviours** | | | | **Experienced suicidal thoughts and behaviours** | | | |
| --- | --- | --- | --- | --- | --- | --- | --- | --- | --- | --- | --- | --- | --- | --- | --- | --- | --- | --- | --- | --- | --- | --- | --- |
|  |  |  | **Total sample** | | | **Men** | | | **Women** | | | **Yes vs. No** | | | | **Men vs. Women** | | | | **Men vs. Women** | | | |
| **Response based on stressful life experience** |  | **No** | | **Yes** | **No** | | **Yes** | **No** | | **Yes** | **χ2** | | ***P*** | **CV^a^** | **HB^b^** | **χ2** | ***P*** | **CV^a^** | **HB^b^** | **χ2** | ***P*** | **CV^a^** | **HB^b^** |
|  | n | 1,630 | | 1,388 | 562 | | 470 | 1,068 | | 918 |  | |  |  |  |  |  |  |  |  |  |  |  |
| *Became aggressive* | % Yes | 10.7 | | 22.1 | 14.6 | | 27.7 | 8.7 | | 19.3 | 72.21 | | <.001 | .16 | <.001 | 13.30 | <.001 | .09 | <.001 | 12.74 | <.001 | .10 | <.01 |
| *Bossy/ inflexible/ angry* | % Yes | 29.3 | | 41.8 | 28.3 | | 37.6 | 29.8 | | 44.0 | 51.79 | | <.001 | .13 | <.001 | 0.39 | .57 | .02 | 1.00 | 5.21 | .02 | .06 | .27 |
| *Eat more or less* | % Yes | 57.6 | | 75.6 | 47.5 | | 66.8 | 62.9 | | 80.1 | 107.83 | | <.001 | .19 | <.001 | 35.73 | <.001 | .15 | <.001 | 29.60 | <.001 | .15 | <.001 |
| *Spiritual activity* | % Yes | 31.8 | | 29.5 | 31.5 | | 27.7 | 32.0 | | 30.5 | 1.86 | | .17 | .03 | <.001 | 0.05 | .87 | .01 | 1.00 | 1.21 | .27 | .03 | 1.00 |
| *Got professional help* | % Yes | 23.9 | | 47.2 | 25.1 | | 47.7 | 23.2 | | 46.9 | 180.14 | | <.001 | .24 | <.001 | 0.72 | .40 | .02 | 1.00 | 0.07 | .79 | .01 | 1.00 |
| *Increased tobacco/alcohol/drugs* | % Yes | 22.4 | | 39.2 | 26.8 | | 43.3 | 20.0 | | 37.1 | 101.19 | | <.001 | .18 | <.001 | 9.83 | .002 | .08 | .02 | 5.03 | .03 | .06 | .27 |
| *Isolated self* | % Yes | 41.4 | | 75.8 | 43.6 | | 75.2 | 40.2 | | 76.2 | 363.66 | | <.001 | .35 | <.001 | 1.71 | .19 | .03 | .96 | 0.17 | .68 | .01 | 1.00 |
| *Overdo activities* | % Yes | 24.2 | | 30.3 | 20.3 | | 25.3 | 26.3 | | 32.9 | 14.12 | | <.001 | .07 | <.001 | 7.23 | .007 | .07 | .06 | 8.38 | .004 | .08 | .05 |
| *Sleep too much/too little* | % Yes | 63.8 | | 83.1 | 60.9 | | 80.6 | 65.3 | | 84.3 | 140.61 | | <.001 | .21 | <.001 | 3.14 | .08 | .04 | .53 | 2.99 | .08 | .05 | .84 |
| *Spend time with friends/loved ones* | % Yes | 37.3 | | 24.3 | 32.7 | | 22.5 | 39.7 | | 25.2 | 59.29 | | <.001 | .14 | <.001 | 7.63 | .006 | .07 | .05 | 1.20 | .27 | .03 | 1.00 |
| *Work less/more* | % Yes | 29.0 | | 45.1 | 31.3 | | 42.3 | 27.8 | | 46.5 | 83.74 | | <.001 | .17 | <.001 | 2.20 | .14 | .04 | .83 | 2.19 | .14 | .04 | 1.00 |
| *Take more risks* | % Yes | 16.6 | | 31.4 | 20.8 | | 35.5 | 14.4 | | 29.3 | 91.10 | | <.001 | .17 | <.001 | 10.93 | .001 | .08 | .01 | 5.53 | .02 | .06 | .24 |
| *Talk to someone about feelings* | % Yes | 71.7 | | 66.9 | 64.5 | | 66.4 | 75.5 | | 67.1 | 8.25 | | <.004 | .05 | .01 | 22.15 | <.001 | .12 | <.001 | 0.08 | .77 | .01 | 1.00 |
| *Talk to someone for advice* | % Yes | 56.2 | | 58.4 | 50.4 | | 56.6 | 59.3 | | 59.4 | 1.49 | | .22 | .02 | .34 | 11.99 | .001 | .09 | <.01 | 0.98 | .32 | .03 | 1.00 |
| *Do nothing* | % Yes | 19.7 | | 33.2 | 23.7 | | 33.0 | 17.6 | | 33.3 | 71.92 | | <.001 | .15 | <.001 | 8.66 | .003 | .07 | .03 | 0.01 | .92 | .00 | 1.00 |
| *Other* | % Yes | 12.6 | | 17.9 | 13.0 | | 16.7 | 12.3 | | 18.4 | 16.45 | | <.001 | .07 | <.001 | 0.16 | .69 | .01 | 1.00 | 0.64 | .42 | .02 | 1.00 |

1. Cramer’s V
2. Holm-Bonferroni correction
