# Supplementary material for: Suicidal Thoughts and Behaviors and Their Associations With Transitional Life Events in Men and Women: Findings From an International Web-Based Sample
Source: JMIR Ment Health. 2020 Sep 11;7(9):e18383. doi: 10.2196/18383 (PMC7519425; doi:10.2196/18383)
Supplement: Multimedia Appendix 2 [file mental_v7i9e18383_app2.docx]

Multimedia Appendix 2. *Participant socio-demographics by country*

|  | | **Australia** | | **Canada** | | **New Zealand** | | **United Kingdom** | | **United States** | |
| --- | --- | --- | --- | --- | --- | --- | --- | --- | --- | --- | --- |
| **Variable** |  | **n** | **%** | **n** | **%** | **n** | **%** | **n** | **%** | **n** | **%** |
| **Gender** | N | 3349 | 31.1 | 1888 | 17.5 | 1752 | 16.3 | 1938 | 18.0 | 1838 | 17.1 |
|  | *Male* | 1282 | 38.3 | 791 | 41.9 | 697 | 39.8 | 762 | 39.3 | 769 | 41.8 |
|  | *Female* | 2067 | 61.7 | 1097 | 58.1 | 1055 | 60.2 | 1176 | 60.7 | 1069 | 58.2 |
| **Age (years)** | N | 3345 | 31.1 | 1886 | 17.5 | 1751 | 16.3 | 1934 | 18.0 | 1837 | 17.1 |
|  | *16 to 24* | 791 | 23.6 | 227 | 12.0 | 604 | 34.5 | 511 | 26.4 | 557 | 30.3 |
|  | *25 to 44* | 1010 | 30.2 | 308 | 16.3 | 378 | 21.6 | 476 | 24.6 | 272 | 14.8 |
|  | *45 to 64* | 1015 | 30.3 | 863 | 45.8 | 540 | 30.8 | 642 | 33.2 | 565 | 30.8 |
|  | *65+* | 529 | 15.8 | 488 | 25.9 | 229 | 13.1 | 305 | 15.8 | 443 | 24.1 |
| **Rural/ remote** | N | 3335 | 31.2 | 1874 | 17.5 | 1742 | 16.3 | 1927 | 18.0 | 1825 | 17.1 |
|  | *No* | 3026 | 90.7 | 1726 | 92.1 | 1701 | 97.6 | 1837 | 95.3 | 1700 | 93.2 |
|  | *Yes* | 309 | 9.3 | 148 | 7.9 | 41 | 2.4 | 90 | 4.7 | 125 | 6.8 |
| **Education, employment and training (EET) status** | N | 3113 | 31.1 | 1730 | 17.3 | 1637 | 16.4 | 1809 | 18.1 | 1709 | 17.1 |
|  | *No (NEET)* | 684 | 22.0 | 744 | 43.0 | 276 | 16.9 | 483 | 26.7 | 591 | 34.6 |
|  | *Yes (EET)* | 2429 | 78.0 | 986 | 57.0 | 1361 | 83.1 | 1326 | 73.3 | 1118 | 65.4 |
| **Living arrangements** | N | 2244 | 31.5 | 1198 | 16.8 | 1145 | 16.1 | 1251 | 17.6 | 1277 | 17.9 |
|  | *Live alone* | 399 | 17.8 | 276 | 23.0 | 167 | 14.6 | 217 | 17.3 | 275 | 21.5 |
|  | *Do not live alone* | 1845 | 82.2 | 922 | 77.0 | 978 | 85.4 | 1034 | 82.7 | 1002 | 78.5 |
| **Language Background** | N | 2245 | 30.0 | 1305 | 17.4 | 1225 | 16.4 | 1358 | 18.1 | 1351 | 18.1 |
|  | *English* | 1858 | 82.8 | 1068 | 81.8 | 930 | 75.9 | 1178 | 86.7 | 937 | 69.4 |
|  | *Language Background Other Than English (LBOTE)* | 387 | 17.2 | 237 | 18.2 | 295 | 24.1 | 180 | 13.3 | 414 | 30.6 |
| **Sexual orientation** | N | 2245 | 31.5 | 1198 | 16.8 | 1145 | 16.1 | 1251 | 17.6 | 1277 | 17.9 |
|  | *Heterosexual* | 1871 | 83.3 | 1020 | 85.1 | 917 | 80.1 | 1021 | 81.6 | 1094 | 85.7 |
|  | *LGBTQIA* | 374 | 16.7 | 178 | 14.9 | 228 | 19.9 | 230 | 18.4 | 183 | 14.3 |
| ***Social connectedness*** |  | N | Mean (SD) | N | Mean (SD) | N | Mean (SD) | N | Mean (SD) | N | Mean (SD) |
|  | *Intimate Bonds (IBM)* | 2546 | 9.2 (2.7) | 1379 | 9.3 (2.6) | 1319 | 9.0 (2.7) | 1460 | 9.2  (2.7) | 1435 | 9.6  (2.7) |
|  | *Social Support (SSSC)* | 2533 | 25.5 (9.8) | 1374 | 25.5 (9.6) | 1312 | 24.9 (9.3) | 1454 | 25.5 (9.6) | 1429 | 26.0  (9.3) |
| ***Transitional Life Event*** | N | 2853 | 31.6 | 1546 | 17.1 | 1461 | 16.2 | 1604 | 17.8 | 1571 | 17.4 |
|  | *Became a parent for the first time (Yes)* | 53 | 1.9 | 9 | 0.6 | 27 | 1.8 | 29 | 1.8 | 27 | 1.7 |
|  | *Finished high school/secondary school (Yes)* | 128 | 4.5 | 64 | 4.1 | 115 | 7.9 | 186 | 7.9 | 113 | 7.2 |
|  | *Started university/college (Yes)* | 210 | 7.4 | 80 | 5.2 | 143 | 9.8 | 197 | 9.8 | 169 | 10.8 |
|  | *Started a new job (Yes)* | 717 | 25.1 | 279 | 18.0 | 401 | 27.4 | 386 | 24.0 | 370 | 23.5 |
|  | *Suddenly or unexpectedly become unemployed (Yes)* | 245 | 8.6 | 158 | 10.2 | 157 | 10.7 | 98 | 6.1 | 135 | 8.6 |
|  | *Retired (Yes)* | 138 | 4.8 | 155 | 10.0 | 68 | 4.7 | 98 | 6.1 | 139 | 8.8 |
|  | *Relationship breakdown (Yes)* | 610 | 21.4 | 312 | 20.2 | 416 | 28.5 | 311 | 19.4 | 424 | 27.0 |
|  | *Transitional life event perceived as stressful* | 995 | 72.0 | 491 | 74.4 | 579 | 72.3 | 514 | 65.4 | 563 | 67.9 |
|  | *Transitional life event not perceived as stressful* | 387 | 28.0 | 169 | 25.6 | 222 | 27.7 | 272 | 34.6 | 266 | 32.1 |
