# Supplementary material for: Suicidal Thoughts and Behaviors and Their Associations With Transitional Life Events in Men and Women: Findings From an International Web-Based Sample
Source: JMIR Ment Health. 2020 Sep 11;7(9):e18383. doi: 10.2196/18383 (PMC7519425; doi:10.2196/18383)
Supplement: Multimedia Appendix 3 [file mental_v7i9e18383_app3.docx]

Multimedia Appendix 3. *Frequency and experience of suicidal thoughts and behaviours*

| **PSFS items** |  | **Total Sample** | **Men** | **Women** | **χ2 (Men vs Women)** | ***P*** | **CV^a^** | **HB^b^** |
| --- | --- | --- | --- | --- | --- | --- | --- | --- |
| **In the past 12 months have you…** | N | 8,708 | 3,491 | 5,217 |  |  |  |  |
| *Felt that life is hardly worth living?* | (% Yes) | 30.8 | 27.2 | 33.3 | 37.09 | <.001 | .07 | <.001 |
| *Thought that you really would be better off dead?* | (% Yes) | 25.1 | 21.7 | 27.4 | 36.14 | <.001 | .06 | <.001 |
| *Thought about taking your own life?* | (% Yes) | 23.8 | 22.4 | 24.7 | 6.13 | .01 | .01 | .01 |
| *Made plans to take your own life?* | (% Yes) | 7.8 | 6.5 | 8.7 | 13.79 | <.001 | .04 | <.001 |
| *Attempted to take your own life?* | (% Yes) | 3.0 | 1.9 | 3.8 | 24.52 | <.001 | .05 | <.001 |
| *STB (Total PSFS)* | (% reporting any STB) | 30.7 | 28.3 | 32.3 | 15.87 | <.001 | .04 | <.001 |

1. Cramer’s V
2. Holm-Bonferroni correction
