# Supplementary material for: Suicidal Thoughts and Behaviors and Their Associations With Transitional Life Events in Men and Women: Findings From an International Web-Based Sample
Source: JMIR Ment Health. 2020 Sep 11;7(9):e18383. doi: 10.2196/18383 (PMC7519425; doi:10.2196/18383)
Supplement: Multimedia Appendix 4 [file mental_v7i9e18383_app4.docx]

Multimedia Appendix 4. *Odds and adjusted odds ratios for men and women’s self-reported suicidal thoughts and behaviours by socio-demographic variables including social connection and transitional life events (Men n= 2,667; Women n= 3,826)*

|  | **PSFS** | | | |
| --- | --- | --- | --- | --- |
|  | **Women** | | **Men** | |
|  | **OR [95% CI]** | **Adjusted OR [95% CI]**^a^ | **OR [95% CI]** | **Adjusted OR [95% CI]**^a^ |
| **Socio-demographic characteristics** |  |  |  |  |
| Age-bands (years) *vs 65+* |  |  |  |  |
| *16 - 24* | 2.92 | 4.03 | 2.56 | 2.89 |
|  | [2.46-3.47]^b^ | [2.78-5.85]^b^ | [2.14-3.05]^b^ | [1.96-4.26]^b^ |
| *25 - 44* | 2.05 | 2.71 | 1.83 | 1.97 |
|  | [1.71-2.45]^b^ | [1.89-3.89]^b^ | [1.52-2.20]^b^ | [1.38-2.80]^b^ |
| *45 - 64* | 1.69 | 1.81 | 1.68 | 1.96 |
|  | [1.41-2.03]^b^ | [1.31-2.49]^b^ | [1.41-2.00]^b^ | [1.46-2.64]^b^ |
| *65+* | 1.00 | 1.00 | 1.00 | 1.00 |
| Rural |  |  |  |  |
| *Yes* | 1.08 | 1.35 | 1.03 | 1.04 |
|  | [0.93-1.26] | [0.98-1.85] | [0.84-1.26] | [0.72-1.50] |
| *No* | 1.00 | 1.00 | 1.00 | 1.00 |
| Sexual orientation |  |  |  |  |
| *Lesbian, Gay, Bisexual, Trans, Queer, Intersex, Asexual*  *(LGBTQIA)* | 2.16 | 3.01 | 1.78 | 1.96 |
|  | [1.98-2.35]^b^ | [2.45-3.71]^b^ | [1.59-2.01]^b^ | [1.56-2.46]^b^ |
| *Heterosexual* | 1.00 | 1.00 | 1.00 | 1.00 |
| Language background other than English (LBOTE) |  |  |  |  |
| *Yes* | 0.93 | 0.64 | 0.85 | 0.67 |
|  | [0.83-1.04] | [0.51-0.80]^b^ | [0.72-0.995]^c^ | [0.51-0.88]^b^ |
| *No* | 1.00 | 1.00 | 1.00 | 1.00 |
| Employment, education or training |  |  |  |  |
| *No (NEET)* | 1.09 | 1.67 | 1.04 | 1.27 |
|  | [0.99-1.21] | [1.31-2.15]^b^ | [0.92-1.16] | [0.98-1.63] |
| *Yes (EET)* | 1.00 | 1.00 | 1.00 | 1.00 |
| Living arrangements |  |  |  |  |
| *Live alone* | 1.01  [0.90-1.13] | 1.38  [1.11-1.71]^b^ | 1.28  [1.12-1.46]^b^ | 1.27  [0.99-1.61] |
|  |  |  |  |  |
| *Live with others* |  | 1.00 |  | 1.00 |
| Social connectedness |  |  |  |  |
| *Intimate Bonds (IBM)* | - | 0.98 | - | 0.96 |
|  |  | [0.97-0.99]^b^ |  | [0.95-0.98]^b^ |
| *Social Support (SSSC)* | - | 0.78 | - | 0.87 |
|  |  | [0.75-0.81]^b^ |  | [0.83-0.90]^b^ |
| Transitional life events |  |  |  |  |
| *Became a parent for the first time* | 0.92 | 0.66 | 0.75 | 0.30 |
|  | [0.65-1.30] | [0.31-1.41] | [0.46-1.25] | [0.10-0.86]^c^ |
| *Finished high school/secondary school* | 1.59 | 0.96 | 1.45 | 1.07 |
|  | [1.43-1.78]^b^ | [0.69-1.33] | [1.20-1.75]^b^ | [0.65-1.77] |
| *Started university/college* | 1.38 | 0.83 | 1.36 | 0.81 |
|  | [1.24-1.54]^b^ | [0.62-1.10] | [1.15-1.61]^b^ | [0.54-1.21] |
| *Started a new job* | 1.21 | 0.80 | 1.22 | 0.73 |
|  | [1.11-1.31]^b^ | [0.65-1.00]^c^ | [1.08-1.37]^b^ | [0.55-0.97]^c^ |
| *Suddenly or unexpectedly become unemployed* | 1.51 | 1.24 | 1.80 | 1.63 |
|  | [1.35-1.68]^b^ | [0.93-1.66] | [1.58-2.05]^b^ | [1.16-2.29]^b^ |
| *Retired* | 0.64 | 0.44 | 0.72 | 0.52 |
|  | [0.51-0.80]^b^ | [0.29-0.68]^b^ | [0.57-0.93]^b^ | [0.33-0.82]^b^ |
| *Relationship breakdown* | 1.88 | 0.93 | 2.11 | 1.45 |
|  | [1.74-2.03]^b^ | [0.73-1.18] | [1.91-2.34]^b^ | [1.06-1.98]^c^ |
| Transitional life event perceived as stressful | 1.96 | 1.55 | 2.71 | 1.38 |
|  | [1.71-2.26]^b^ | [1.34-1.79]^b^ | [2.22-3.31]^b^ | [1.15-1.67]^b^ |
| **Nagelkerke R^2^** |  | 0.31 |  | 0.24 |

^a^ adjusted for all variables.

^b^ significant at a 99% confidence level.

^c^ significant at a 95% confidence level.
