# Supplementary material for: Suicidal Thoughts and Behaviors and Their Associations With Transitional Life Events in Men and Women: Findings From an International Web-Based Sample
Source: JMIR Ment Health. 2020 Sep 11;7(9):e18383. doi: 10.2196/18383 (PMC7519425; doi:10.2196/18383)
Supplement: Multimedia Appendix 5 [file mental_v7i9e18383_app5.docx]

Multimedia Appendix 5. *Frequency of men and women’s responses based on stressful transitional life event experience for participants who did and did not report suicidal thoughts and behaviours (PSFS)*

|  | | | **Suicidal thoughts and behaviours PSFS** | | | | | | | | | **Suicidal thoughts and behaviours for total sample** | | | | **Did not experience suicidal thoughts and behaviours** | | | | **Experienced suicidal thoughts and behaviours** | | | |
| --- | --- | --- | --- | --- | --- | --- | --- | --- | --- | --- | --- | --- | --- | --- | --- | --- | --- | --- | --- | --- | --- | --- | --- |
|  |  |  | **Total sample** | | | **Men** | | | **Women** | | | **Yes vs. No** | | | | **Men vs. Women** | | | | **Men vs. Women** | | | |
| **Response based on stressful life experience** |  | **No** | | **Yes** | **No** | | **Yes** | **No** | | **Yes** | **χ2** | | ***P*** | **CV^a^** | **HB^b^** | **χ2** | ***P*** | **CV^a^** | **HB^b^** | **χ2** | ***P*** | **CV^a^** | **HB^b^** |
|  | n | 1,630 | | 1,388 | 562 | | 470 | 1,068 | | 918 |  | |  |  |  |  |  |  |  |  |  |  |  |
| *Became aggressive* | % Yes | 10.7 | | 22.1 | 14.6 | | 27.7 | 8.7 | | 19.3 | 72.21 | | <.001 | .16 | <.001 | 13.30 | <.001 | .09 | <.001 | 12.74 | <.001 | .10 | <.01 |
| *Bossy/ inflexible/ angry* | % Yes | 29.3 | | 41.8 | 28.3 | | 37.6 | 29.8 | | 44.0 | 51.79 | | <.001 | .13 | <.001 | 0.39 | .57 | .02 | 1.00 | 5.21 | .02 | .06 | .27 |
| *Eat more or less* | % Yes | 57.6 | | 75.6 | 47.5 | | 66.8 | 62.9 | | 80.1 | 107.83 | | <.001 | .19 | <.001 | 35.73 | <.001 | .15 | <.001 | 29.60 | <.001 | .15 | <.001 |
| *Spiritual activity* | % Yes | 31.8 | | 29.5 | 31.5 | | 27.7 | 32.0 | | 30.5 | 1.86 | | .17 | .03 | <.001 | 0.05 | .87 | .01 | 1.00 | 1.21 | .27 | .03 | 1.00 |
| *Got professional help* | % Yes | 23.9 | | 47.2 | 25.1 | | 47.7 | 23.2 | | 46.9 | 180.14 | | <.001 | .24 | <.001 | 0.72 | .40 | .02 | 1.00 | 0.07 | .79 | .01 | 1.00 |
| *Increased tobacco/alcohol/drugs* | % Yes | 22.4 | | 39.2 | 26.8 | | 43.3 | 20.0 | | 37.1 | 101.19 | | <.001 | .18 | <.001 | 9.83 | .002 | .08 | .02 | 5.03 | .03 | .06 | .27 |
| *Isolated self* | % Yes | 41.4 | | 75.8 | 43.6 | | 75.2 | 40.2 | | 76.2 | 363.66 | | <.001 | .35 | <.001 | 1.71 | .19 | .03 | .96 | 0.17 | .68 | .01 | 1.00 |
| *Overdo activities* | % Yes | 24.2 | | 30.3 | 20.3 | | 25.3 | 26.3 | | 32.9 | 14.12 | | <.001 | .07 | <.001 | 7.23 | .007 | .07 | .06 | 8.38 | .004 | .08 | .05 |
| *Sleep too much/too little* | % Yes | 63.8 | | 83.1 | 60.9 | | 80.6 | 65.3 | | 84.3 | 140.61 | | <.001 | .21 | <.001 | 3.14 | .08 | .04 | .53 | 2.99 | .08 | .05 | .84 |
| *Spend time with friends/loved ones* | % Yes | 37.3 | | 24.3 | 32.7 | | 22.5 | 39.7 | | 25.2 | 59.29 | | <.001 | .14 | <.001 | 7.63 | .006 | .07 | .05 | 1.20 | .27 | .03 | 1.00 |
| *Work less/more* | % Yes | 29.0 | | 45.1 | 31.3 | | 42.3 | 27.8 | | 46.5 | 83.74 | | <.001 | .17 | <.001 | 2.20 | .14 | .04 | .83 | 2.19 | .14 | .04 | 1.00 |
| *Take more risks* | % Yes | 16.6 | | 31.4 | 20.8 | | 35.5 | 14.4 | | 29.3 | 91.10 | | <.001 | .17 | <.001 | 10.93 | .001 | .08 | .01 | 5.53 | .02 | .06 | .24 |
| *Talk to someone about feelings* | % Yes | 71.7 | | 66.9 | 64.5 | | 66.4 | 75.5 | | 67.1 | 8.25 | | <.004 | .05 | .01 | 22.15 | <.001 | .12 | <.001 | 0.08 | .77 | .01 | 1.00 |
| *Talk to someone for advice* | % Yes | 56.2 | | 58.4 | 50.4 | | 56.6 | 59.3 | | 59.4 | 1.49 | | .22 | .02 | .34 | 11.99 | .001 | .09 | <.01 | 0.98 | .32 | .03 | 1.00 |
| *Do nothing* | % Yes | 19.7 | | 33.2 | 23.7 | | 33.0 | 17.6 | | 33.3 | 71.92 | | <.001 | .15 | <.001 | 8.66 | .003 | .07 | .03 | 0.01 | .92 | .00 | 1.00 |
| *Other* | % Yes | 12.6 | | 17.9 | 13.0 | | 16.7 | 12.3 | | 18.4 | 16.45 | | <.001 | .07 | <.001 | 0.16 | .69 | .01 | 1.00 | 0.64 | .42 | .02 | 1.00 |

1. Cramer’s V
2. Holm-Bonferroni correction
